# Embolic Burden and Echocardiographic Predictors in a Real-World Cohort of Infective Endocarditis: A 15-Year Single-Center Retrospective Study

**DOI:** 10.3390/jcm15103769

**Published:** 2026-05-14

**Authors:** Călin Pop, Lucian Liviu Pop, Maria Rebeca Petruș, Andreea Ioana Talpos, Roxana Hodas, Lavinia Pop, Iulia Pop

**Affiliations:** 1 Department of Cardiology, “Constantin Opriş” Emergency County Hospital, Str. George Cosbuc Nr. 31, CP 430130 Baia Mare, Romania; secr.cardiologie@sjbm.ro (M.R.P.); cardiologie@sjbm.ro (A.I.T.); roxana.hodas@yahoo.ro (R.H.); laviniapop10@yahoo.com (L.P.); dr_iulia_muresan@yahoo.com (I.P.); 2 Faculty of Nursing and Health Sciences, University of Medicine and Pharmacy “Iuliu Hațieganu”, Str. Louis Pasteur Nr. 4, CP 400349 Cluj-Napoca, Romania; pop.lucianliviu@yahoo.com

**Keywords:** infective endocarditis, embolic events, vegetation mobility, echocardiography, embolic risk stratification, vegetation size, transesophageal echocardiography, prognostic factors

## Abstract

**Background/Objectives:** Systemic embolization is a common and serious complication of infective endocarditis (IE). This study evaluated the association between vegetation morphology and embolic events and assessed whether echocardiographic parameters provide incremental discriminatory value beyond clinical variables. **Methods:** We conducted a retrospective cohort study including 164 consecutive adults hospitalized with definite IE between 2011 and 2025 at a regional referral center. Vegetation presence, size, and mobility were assessed using transthoracic (TTE) and transesophageal echocardiography (TEE), according to clinical indication. The primary endpoint was overall in-hospital embolic burden, including embolic events present at admission, occurring during hospitalization, or incidentally detected during diagnostic work-up. Associations were analyzed using univariate and multivariable logistic regression, and model discrimination was evaluated using receiver operating characteristic (ROC) analysis. **Results:** Embolic events occurred in 96 patients (58.5%). Vegetations were identified in 68.3% of patients and were more frequent among those with embolization (78.1% vs. 54.4%). Mobile vegetations were more common in patients with embolic events (77.1% vs. 27.9%, *p* < 0.001), as were vegetations > 10 mm (61.5% vs. 38.2%, *p* = 0.006). Compared with non-mobile vegetations ≤ 10 mm, mobile vegetations ≤ 10 mm were associated with higher odds of embolization (OR 5.4), and mobile vegetations > 10 mm showed a similar association (OR 7.14). In multivariable analysis, vegetation mobility remained independently associated with embolic events. The clinical model demonstrated moderate discrimination (area under the curve [AUC] 0.71), which improved with the addition of vegetation mobility (AUC 0.81; *p* = 0.005) and size > 10 mm (AUC 0.79; *p* = 0.016), with no significant difference between the enhanced models. **Conclusions:** Both vegetation mobility and size > 10 mm were associated with overall in-hospital embolic burden and may provide complementary information for embolic risk stratification. These findings should be considered exploratory and require confirmation in prospective studies with standardized imaging and validation procedures.

## 1. Introduction

Infective endocarditis (IE) is an uncommon but highly morbid disease, with an incidence ranging from 1.5 to 11.6 cases per 100,000 persons per year, with persistently high mortality. Contemporary cohorts report in-hospital mortality of approximately 15–20%, while longer-term mortality may reach 30–40% in high-risk populations [1,2]. Despite advances in antimicrobial therapy, surgical management, and multimodal imaging, IE remains a life-threatening condition frequently complicated by heart failure, uncontrolled infection, and embolic events, all of which significantly impact prognosis and healthcare utilization [2,3]. Systemic embolization is one of the most common and clinically significant complications of IE. Observational cohorts report embolic events (EE) in approximately 20–50% of patients, with 10–25% already present at diagnosis [1,2]. In the European Society of Cardiology (ESC) EURObservational Research Programme (EORP) European Endocarditis (EURO-ENDO) registry, which includes nearly 3000 patients, embolic events occurred in 20.6% and were independently associated with vegetation presence and *Staphylococcus aureus* infection [2].

Importantly, embolization is often clinically silent; brain and splenic emboli may remain asymptomatic in up to 20–50% of patients and are frequently detected only through systematic computed tomography (CT) or magnetic resonance imaging (MRI) [4,5,6]. This hidden embolic burden has important implications for management and surgical timing. Embolic risk is strongly time-dependent. Prospective studies show that the risk of embolization is highest around the initiation of effective antibiotic therapy and declines substantially during the first two weeks, although residual risk persists as long as vegetations remain present [7]. These dynamics support early risk stratification and, in selected patients, early surgery aimed at embolic prevention. The 2023 ESC Guidelines integrated imaging-detected embolic dissemination into the diagnostic framework and reinforced the role of multimodal imaging [3]. Brain and whole-body imaging (CT, positron emission tomography—PET/CT, MRI) are recommended, particularly in symptomatic patients, and may contribute diagnostic minor criteria in selected cases [3]. Nevertheless, echocardiography remains the cornerstone for both diagnosis and embolic risk assessment [3,8]. Transthoracic echocardiography (TTE) and, especially, transesophageal echocardiography (TEE) provide detailed evaluations of vegetation morphology, valvular destruction, and perivalvular extension, forming the basis for dynamic risk stratification.

Vegetations are typically described as oscillating masses attached to the low-pressure side of cardiac valves [3,8]. The emboligenic phenotype includes large size, marked mobility, and mitral valve involvement, particularly in staphylococcal IE. Historically, vegetation size has served as a pragmatic marker of embolic risk. A meta-analysis involving more than 6500 patients demonstrated that vegetations > 10 mm were associated with increased embolic events (OR ≈ 2.28) and mortality (OR ≈ 1.63) compared to smaller vegetations [9]. Registry analyses further suggest that the prognostic impact of vegetation size interacts with treatment strategy and surgical timing [10]. However, vegetation mobility appears to confer additional risk beyond size. In a prospective multicenter study, Thuny et al. demonstrated that both vegetation length and mobility were independently associated with new embolic events and mortality [8]. More recent analyses confirm that combining echocardiographic parameters with clinical variables improves embolic risk prediction [11]. Accordingly, the 2023 ESC Guidelines emphasize large and mobile vegetations as markers of increased embolic risk and potential indications for early surgical evaluation [3].

TEE improves diagnostic sensitivity for vegetations to approximately 85–90% and is particularly valuable for detecting abscesses and perivalvular complications [3]. In patients with *Staphylococcus aureus*, TEE detects endocardial involvement in approximate 86% of cases, compared to only 21% by TTE [12]. However, reliance on vegetation size thresholds is limited by measurement variability. Recent studies have demonstrated significant interobserver variability in vegetation diameter assessment, cautioning against strict reliance on size cut-offs as surgical triggers [13]. Three-dimensional TEE may further improve morphological characterization and refine embolic prediction compared with conventional two-dimensional imaging [14,15]. Several predictive models have been proposed to quantify embolic risk. The French embolic risk calculator incorporates age, diabetes, atrial fibrillation, prior embolism, vegetation length, and *Staphylococcus aureus* infection and has undergone external validation [16,17]. However, guideline discussions emphasize that predictive models may perform variably across real-world populations [3].

Although international registries such as EURO-ENDO have provided robust epidemiological data, Eastern European cohorts remain comparatively limited [2]. Romanian studies describe IE in centers without on-site cardiac surgery, emerging intravenous drug use (IVDU)-associated phenotypes, and contemporary microbiological profiles in tertiary centers [18,19,20]. However, structured echocardiographic analyses of embolic risk in this region remain scarce. Therefore, we conducted a 15-year retrospective cohort study to investigate the association between echocardiographic vegetation characteristics and in-hospital embolic burden in IE. The study aimed to (1) evaluate the association between vegetation morphology—particularly mobility and size—and overall in-hospital embolic burden; (2) determine whether echocardiographic parameters provide incremental discriminatory value beyond clinical variables; and (3) contextualize these findings relative to international registries and contemporary Romanian cohorts [2,3,11,18,19,20,21].

## 2. Methods

### 2.1. Study Design and Setting

This retrospective, single-center, observational, cohort study was conducted in the Department of Cardiology and Cardiovascular Intensive Care at the “Dr. Constantin Opriș” Emergency County Hospital, Baia Mare, Romania, a regional secondary/tertiary referral center. Consecutive adult patients hospitalized with definitive IE between 1 January 2011, and 31 December 2025, were included. The Institutional Review Ethics Committee of the “Dr. Constantin Opriș” Emergency County Hospital approved the study protocol on 2 February 2026 (decision no. 4979). Due to the retrospective design and the use of anonymized routinely collected clinical data, the requirement for individual informed consent was waived in accordance with institutional regulations. During manuscript preparation, the authors utilized ChatGPT Plus AI tools for graphic abstract creation. The study followed the STROBE recommendations for observational studies [22].

### 2.2. Participants and Eligibility Criteria

All consecutive adult patients (≥18 years) hospitalized with suspected IE during the study period were screened. Patients were eligible for inclusion if they met the criteria for definite IE according to the modified Duke criteria, supported by clinical, microbiological, and echocardiographic data. Diagnostic classification incorporated the 2023 ESC guideline update, which integrates advanced imaging findings into the diagnostic framework [3,23]. According to ESC definitions, patients were categorized as having native valve IE (NVE), prosthetic valve IE (PVE), or cardiac implantable electronic device (CIED)-related IE [3]. Both left-sided and right-sided IE cases were included. Exclusion criteria comprised possible or rejected IE diagnosis, hospital stay < 24 h with insufficient clinical data, and isolated catheter-related right-sided infections without essential diagnostic documentation.

### 2.3. Data Collection

Clinical, microbiological, and imaging data were extracted from electronic medical records, microbiology databases, echocardiography archives, and discharge summaries.

Variables were organized into predefined domains:

- Clinical variables: age, sex, comorbidity burden, atrial fibrillation, acute heart failure at admission, sepsis, and septic shock.
- Microbiological variables: blood culture results, causative pathogen, *Staphylococcus aureus* infection, and culture-negative IE.
- Echocardiographic assessment: All patients underwent TTE, whereas TEE was performed when clinically indicated, particularly in cases with inconclusive TTE findings, suspected complications, or extended clinical suspicion of infective endocarditis. Echocardiographic assessment was generally performed at admission or early during hospitalization, according to routine clinical practice. Due to the retrospective design and extended inclusion period, detailed data regarding the exact proportion and timing of the TEE were not consistently available. In patients who underwent both examinations, TEE findings were considered the reference standard for vegetation detection and characterization. The use of TEE increased progressively over the study period, becoming more systematic after 2016–2017, in parallel with evolving guideline recommendations and local imaging availability. To address potential temporal bias, a sensitivity analysis compared patients from earlier and later study periods.
- Vegetation assessment: Vegetations were considered present when identified on TTE or TEE. Echocardiographic evaluation included vegetation presence, maximal vegetation length, vegetation mobility, valve involvement, severity of valvular regurgitation, and perivalvular complications. A vegetation was defined as an oscillating intracardiac mass attached to a valve or endocardial structure, consistent with ESC imaging criteria [3]. Vegetation mobility was defined as independent oscillatory motion of the vegetation body relative to the valve plane, exceeding passive valve movement [8,24]. Mobility was assessed qualitatively and recorded as a binary variable (mobile vs. non-mobile), consistent with prior echocardiographic studies. Interpretation was based on consensus between two experienced echocardiographers within the routine clinical workflow. When both TTE and TEE were available, TEE findings were preferentially used due to their superior sensitivity and spatial resolution [3,8,25]. Given the retrospective design, formal interobserver variability analysis and systematic blinding to clinical outcomes were not performed.
- Imaging for embolic events: Imaging (CT, MRI, or other modalities) was performed based on clinical indications and was not systematically applied to all patients. The availability and use of advanced imaging increased over time, particularly after 2015. Consequently, detection of embolic events may have been influenced by differences in diagnostic intensity, and the primary endpoint reflects overall in-hospital embolic burden rather than uniformly screened events.

### 2.4. Outcomes

The primary endpoint was the occurrence of in-hospital embolic events. Embolic events were defined as systemic embolization with clinical and/or imaging confirmation during hospitalization. Events were classified according to the affected territory:

- Cerebral embolism—ischemic stroke or imaging-confirmed cerebral infarction.
- Peripheral embolism—splenic, renal, hepatic, mesenteric, or limb arterial embolization.

Events were further categorized as symptomatic or incidentally detected on imaging, in line with definitions used in previous IE cohorts and registries [3,4,5,6,7,8,9,10,11].

Embolic events were classified according to their temporal relationship with hospitalization as the following: (1) present at admission, (2) newly occurring during hospitalization, or (3) incidentally detected during diagnostic work-up. Given the retrospective design, precise timing relative to antibiotic initiation was not consistently available; therefore, embolic events were analyzed as a composite in-hospital endpoint reflecting overall embolic burden, with a supplementary sensitive analysis exploring early versus in-hospital embolization.

Secondary endpoints comprised three categories:

- Clinical complications: acute heart failure, septic shock, cardiogenic shock, and the need for renal replacement therapy.
- Management-related variables: cardiac surgery during the indexed hospitalization and appropriate antimicrobial therapy, defined as empirical and/or targeted antibiotic treatment consistent with microbiological findings and guideline-recommended duration.
- Hospital outcomes: in-hospital mortality and length of hospital stay (median days).

Two investigators independently performed case identification and data verification, with discrepancies resolved by a senior cardiologist through adjudication.

## 3. Statistical Analysis

Continuous variables were reported as mean ± standard deviation (SD) or median with interquartile range (IQR), depending on distribution. Categorical variables were presented as counts and percentages. Comparisons between patients with and without embolic events were performed using Student’s *t*-test or the Mann–Whitney *U* test for continuous variables, and the χ^2^ test or Fisher’s exact test for categorical variables. Variables associated with embolic events in univariate analysis were considered for inclusion in the multivariable logistic regression models. Multivariable models included variables with *p* < 0.10 in univariable analysis, along with clinically relevant covariates. The results were expressed as odds ratios (ORs) with 95% confidence intervals (CIs). Subgroup odds ratios were calculated from contingency tables using predefined reference categories. For subgroup analyses with zero-event cells, odds ratios were estimated using the Haldane–Anscombe correction. Confidence intervals were calculated using standard log-transformation methods, and Fisher’s exact test was used for *p*-value estimation where appropriate.

To assess the incremental prognostic value of echocardiographic parameters, three nested models were constructed:

- Model A: clinical variables only.
- Model B1: clinical variables + vegetation mobility.
- Model B2: clinical variables + vegetation size (>10 mm).

Model discrimination was assessed with receiver operating characteristic (ROC) curves, with calculation of the area under the curve (AUC) and corresponding 95% CI. No internal formal validation, calibration analysis, or decision-analytic assessment was performed; therefore, ROC and AUC findings should be interpreted cautiously. Differences between AUC values were assessed using the DeLong test. A two-sided *p* < 0.05 was considered statistically significant. All the analyses were performed using R software (version 4.4.0; R Foundation for Statistical Computing, Vienna, Austria) [26].

## 4. Results

### 4.1. Study Population

**Figure 1:**
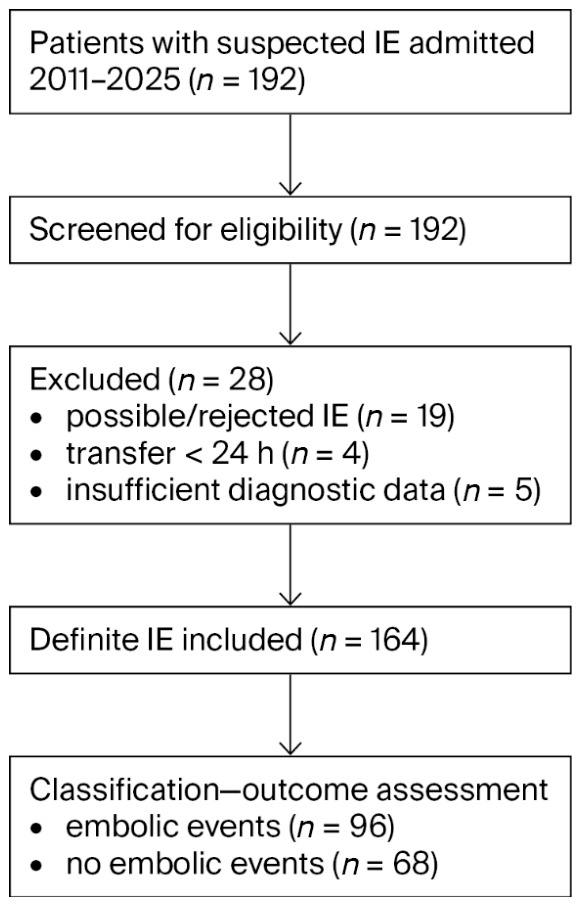
Study Population Selection Flow Diagram. Legend: IE—infective endocarditis; *n*—number.

The study included 164 consecutive patients with definite IE, of whom 96 patients (58.5%) experienced embolic events (Figure 1). Among these patients, echocardiography identified vegetations in 75 cases (78%), whereas 21 patients (22%) had no detectable vegetations. The mean age of the cohort was 64 ± 12.7 years. Patients with embolic events were younger (62 ± 12.2 vs. 66 ± 12.6 years). Men comprised the majority of the cohort; however, women were proportionally more frequent among patients with embolic events. The high rate of embolic events likely reflects the combined contribution of clinically apparent cases and selective imaging, with increased use of advanced imaging modalities in later years of the study.

Regarding comorbidities, diabetes mellitus (DM) was less common among patients with embolic events, suggesting a potential inverse association. In contrast, chronic kidney disease (CKD) ≥ stage 3 was more prevalent among patients with embolic complications, indicating a higher comorbidity burden. Other baseline clinical characteristics, including prosthetic valve endocarditis and microbiological profile, were broadly similar between groups. A detailed comparison of baseline characteristics is presented in Table 1.

### 4.2. Infection Characteristics and Microbiology

Left-sided IE represented the predominant presentation (79.3%), with no significant difference between embolic and non-embolic groups. Right-sided IE was uncommon, with only three cases involving the tricuspid valve. Prosthetic valve endocarditis occurred in 41.5% of patients and showed a non-significant trend toward lower embolic risk. The microbiological distribution was comparable between groups. CIED-related IE was identified in 25 patients (15.2% of the cohort), representing a clinically relevant subgroup predominantly associated with staphylococcal pathogens, consistent with device-related infection mechanisms. Overall, *Staphylococcus aureus* infection was identified in 13.4% of cases and showed no association with embolic events. Culture-negative IE occurred in 47% of patients. The relatively high proportion of culture-negative IE was observed predominantly during the earlier study period and likely reflected frequent antibiotic exposure before referral, delayed presentation, and less systematic microbiological investigation during the initial years of the cohort (Table 1).

### 4.3. Clinical Outcomes

Regarding the primary endpoint, embolic events occurred across multiple vascular territories. Embolic events included those present at admission, as well as those detected or occurring during hospitalization, reflecting the overall in-hospital embolic burden, although precise temporal classification was not consistently available. Most embolic events were already present at admission or identified during early diagnostic evaluation, while approximately 30% occurred during hospitalization. Cerebral circulation accounted for 49% (47 patients) of events, including 30.2% (29 patients) with symptomatic manifestations and 18.8% (18 patients) detected only on imaging. Peripheral arterial territory embolization/circulation accounted for 51% (49 patients), including splenic, renal, hepatic, mesenteric, or limb arteries. Despite the overall balance between cerebral and peripheral events, the anatomical distribution differed according to vegetation phenotypes. Among patients with mobile vegetations > 10 mm, embolic events were predominantly peripheral (35 of 52 events, 67.3%). A similar pattern was observed in patients with mobile vegetations ≤ 10 mm, in whom 59.1% of embolic events involved peripheral territories. In contrast, embolic events in patients without detectable vegetations were exclusively cerebral, including 14 symptomatic strokes and 7 imaging-detected cerebral infarctions among 21 patients. Although the overall frequency of cerebral and peripheral embolic events was comparable, the distribution differed significantly according to vegetation phenotype (chi-square, *p* < 0.001) (Table 2). Secondary clinical outcomes were broadly similar between groups. In-hospital mortality occurred in 37.5% of patients with embolic events compared with 25% in the non-embolic group, although this difference was not statistically significant (*p* = 0.129). The mean length of hospital stay was also comparable between groups (*p* = 0.697) (Table 1).

### 4.4. Echocardiographic Findings

Echocardiographic examination revealed several differences between patients with and without embolic events. Vegetations were identified in 112 patients (68.3%) and were more frequent in the embolic group than in the non-embolic group (78.1% vs. 54.4%). The mitral valve was the most common site of involvement (47 patients, 42%), followed by the aortic valve (44 patients, 39.3%), where tricuspid involvement was rare (3 patients, 2.7%). Multivalvular IE was observed in 13 patients (11.6%). Among patients with vegetations, embolic events occurred in 68.1% (32/47) of mitral valve IE and 61.4% (27/44) of aortic valve IE, with no statistically significant difference between locations (*p* = 0.52). A higher proportion of embolic events was observed in multivalvular IE (83.3%, 15/18). Vegetation morphology differed between groups. Patients with embolic events had larger vegetations (median 14 mm), compared with those without embolization (median 9.5 mm). Using clinically relevant thresholds, vegetations > 10 mm were significantly associated with embolization, occurring in 62.5% of patients with embolic events compared with 20.6% in the non-embolic group (*p* < 0.0001).

Vegetation mobility was assessed using a standardized qualitative definition with consensus interpretation, reflecting routine clinical echocardiographic practice. Vegetations were identified in 112 patients (68.3%) and were significantly more frequent in patients with embolic events than in those without (78.1% vs. 54.4%, *p* = 0.002). Vegetations > 10 mm were also more common in the embolic group (62.5% vs. 20.6%, *p* < 0.001), as was vegetation mobility (77.1% vs. 27.9%, *p* < 0.001). The combined phenotype of mobile vegetations > 10 mm was observed in 55.2% of embolic cases compared with 20.5% of non-embolic cases (*p* < 0.001). These findings indicate a strong association between embolic events and vegetation morphology, particularly mobility and size > 10 mm. A detailed comparison is presented in Table 3. In combined phenotype analyses, embolic risk differed markedly according to vegetation morphology. Compared with non-mobile vegetations ≤ 10 mm, mobile vegetations ≤ 10 mm were associated with substantially higher odds of embolization (OR 49.5; 95% CI 5.5–440; *p* < 0.001), while mobile vegetations > 10 mm showed a similarly strong association (OR 33.4; 95% CI 4.5–250; *p* < 0.001). There was no significant difference between mobile vegetations > 10 mm and mobile vegetations ≤ 10 mm (OR 0.67, *p* = 0.5), suggesting that mobility, rather than size, was the dominant determinant of embolic risk within mobile phenotypes. In valve-specific analyses, mobile vegetations were consistently associated with higher embolic risk. In the aortic subgroup, all embolic events occurred in patients with mobile vegetations; this corresponded to a very large effect estimate with wide confidence intervals, reflecting sparse-data bias and zero-cell correction. In multivalvular involvement, a similar pattern was observed (OR 17.3; 95% CI 0.75–400; *p* = 0.065). Overall, mobile vegetations were strongly associated with embolic events compared with non-mobile vegetations (OR 37; 95% CI 7.96–176.1; *p* < 0.001), as shown in Table 4.

The proportion of patients undergoing TEE and advanced imaging increased substantially during the later study period, reflecting evolving diagnostic strategies and improved local availability. Sensitivity analyses across study periods and embolic timing showed broadly consistent associations between vegetation morphology and embolic burden despite evolving imaging practices and increasing TEE utilization over time.

### 4.5. Univariate Predictors of Embolic Events

To explore potential determinants of embolization, univariate logistic regression analysis was performed (Table 5). Vegetation mobility and vegetation size > 10 mm showed the strongest associations with embolic events. Mobile vegetations were associated with an approximately 8.7-fold higher odds of embolization (OR 8.67; 95% CI 4.26–17.68; *p* < 0.001), while vegetations > 10 mm were associated with a 6.4-fold higher odds (OR 6.43; 95% CI 3.13–13.19; *p* < 0.001). Among clinical variables, CKD ≥ stage 3 and reduced left ventricular ejection fraction (LVEF) < 50% were associated with increased embolic risk, whereas DM showed an inverse association.

### 4.6. Multivariable Logistic Regression Analysis

Variables significantly associated with embolic events in univariate analysis were included in multivariable logistic regression models to assess independent associations (Table 6).

Model A (clinical variables only) showed that CKD ≥ stage 3 and DM were independently associated with embolic events, while LVEF < 50% showed borderline associations. Increasing age was associated with a lower risk of embolic events (OR < 1), indicating a higher embolic burden in younger patients.

In Model B1 (Model A + vegetation mobility), vegetation mobility emerged as a strong independent predictor (OR ~ 3.6). Age retained its inverse association with embolic events, while CKD and LVEF < 50% were no longer significant. Female sex remained independently associated with embolization, and DM retained its inverse association.

In Model B2 (Model A + vegetation size > 10 mm), vegetation size > 10 mm was also independently associated with embolic events (OR ≈ 3.5), with a pattern similar to Model B1: age remained inversely associated, while CKD and reduced LVEF were no longer significant predictors.

**Figure 2:**
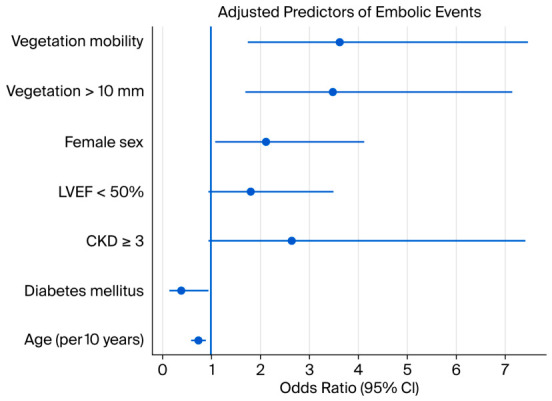
Adjusted Predictors of Embolic Events From Multivariable Logistic Regression. Legend: Points represent adjusted odds ratios (OR), and horizontal lines indicate 95% confidence intervals (CI). The vertical reference line at OR = 1 denotes no association. Vegetation mobility and vegetation size > 10 mm were independently associated with embolic events. Age showed an inverse association (OR < 1), while female sex remained positively associated. Diabetes mellitus was inversely associated with embolization; CKD (chronic kidney disease) ≥ stage 3 and reduced LVEF (left ventricular ejection fraction) < 50% were not independently associated after adjustment.

Overall, the inclusion of echocardiographic parameters attenuated the associations of several clinical variables, suggesting that vegetation morphology is a primary determinant of embolic risk, whereas clinical factors may reflect underlying disease severity rather than direct embolic mechanisms. Adjusted predictors derived from multivariable models are presented in Figure 2.

### 4.7. Model Discrimination and ROC Analysis

**Figure 3:**
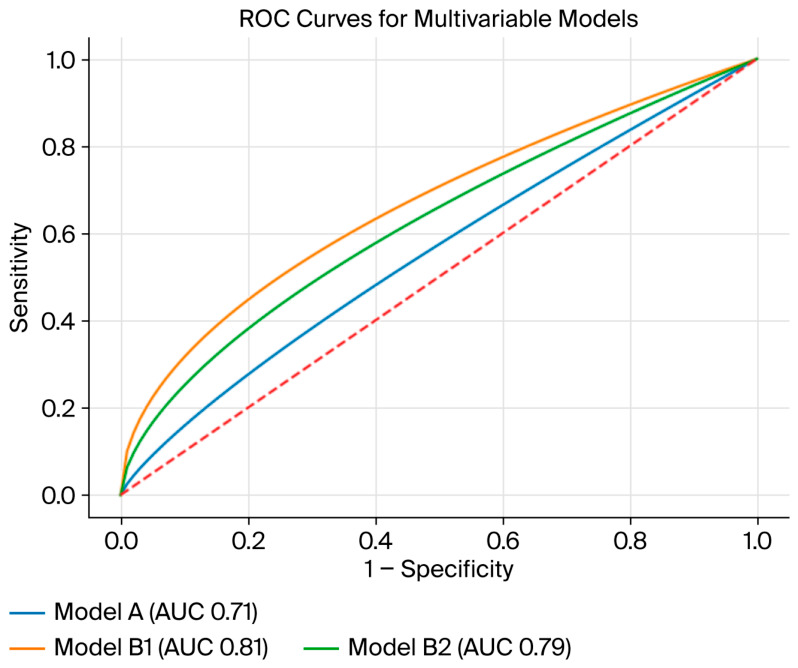
Receiver Operating Characteristic Curves for Multivariable Logistic Regression Models (A, B1, B2). Legend: The diagonal dashed line represents no discrimination (AUC = 0.50). AUC = area under the curve; ROC = receiver operating characteristic. Model A-clinical variables only, Model B1-Model A + vegetation mobility (AUC 0.81, 95% CI 0.75–0.88) and Model B2-Model A + vegetation size > 10 mm (AUC 0.79, 95% CI 0.72–0.86) showed improved discrimination compared with Model A (AUC 0.71, 95% CI 0.63–0.79), with no difference between B1 and B2 (DeLong *p* = 0.256).

The clinical model demonstrated moderate discrimination (AUC 0.712, 95% CI 0.632–0.793). The addition of vegetation mobility improved discrimination (AUC 0.813; 95% CI 0.747–0.880; DeLong *p* = 0.005 vs. Model A). Inclusion of vegetation size > 10 mm also improved discrimination compared with the clinical model (AUC 0.791; 95% CI 0.721–0.861; *p* = 0.016 vs. Model A). There was no statistically significant difference between the mobility-based and size-based models (*p* = 0.256), suggesting that both echocardiographic parameters provide comparable discriminatory information. ROC curves for the three models are shown in Figure 3.

## 5. Discussion

In this 15-year, single-center cohort of 164 patients with definite IE, embolic events occurred in 58.5% of cases, representing a substantial clinical burden. This proportion lies at the upper end of the range reported in contemporary cohorts, where embolization occurs in 20–50% of patients, depending on imaging strategies and diagnosis timing [27,28,29]. This likely reflects the combined effect of referral bias, inclusion of both symptomatic and imaging-detected events, and variability in diagnostic intensity across the study period. Imaging was performed based on clinical indications rather than systematically, and its use increased over time, particularly after 2015, potentially contributing to higher detection of clinically silent embolic events. The extended inclusion period reflects real-world evolution in imaging intensity, microbiological diagnostics, and management strategies, which likely influenced vegetation detection and embolic event ascertainment. Consequently, these results should be interpreted as reflecting overall in-hospital embolic burden in a real-world setting, potentially overestimating absolute event rates rather than a uniformly ascertained incidence. Accordingly, the present analysis should be interpreted as evaluating associations with overall embolic burden rather than a validated prediction of future embolization.

The principal finding of this study is that both vegetation mobility and vegetation size showed incremental discriminatory value beyond clinical variables. The univariate analysis further supports the central role of echocardiographic morphology, with vegetation mobility and vegetation size > 10 mm showing the strongest associations with embolic burden. These findings are consistent with the phenotype-based analyses but should be interpreted as associative given the retrospective design and potential residual confounding. Valve-specific estimates should be interpreted cautiously due to sparse data and the use of zero-cell corrections, resulting in wide confidence intervals. Accordingly, subgroup odds ratios should be interpreted as exploratory associative findings rather than precise quantitative estimates.

In multivariable analysis, both vegetation mobility and vegetation size > 10 mm remained independently associated with embolic burden and improved model discrimination beyond clinical variables alone. ROC analyses similarly demonstrated improved discriminatory performance with the addition of echocardiographic parameters, although these findings should be interpreted cautiously and considered exploratory in the absence of internal validation or calibration procedures.

In this cohort, the primary endpoint combined embolic events present at admission, those incident during hospitalization, and those detected during diagnostic work-up. As embolic risk in IE is strongly time-dependent and declines after initiation of antibiotic therapy, this composite endpoint reflects overall in-hospital embolic burden rather than strictly incident embolization. Consequently, the observed associations between vegetation morphology and embolic events likely reflect an underlying emboligenic phenotype rather than purely time-dependent prognostic relationships. This approach aligns with real-world clinical practice, where baseline imaging is not systematically performed and embolic events may be identified at different stages of evaluation. Future prospective studies incorporating time-to-event analyses are needed to better define the temporal dynamics of embolic risk.

The present study provides a real-world, long-term perspective (2011–2025) from an unselected cohort, including patients with prosthesis valves, CIEDs, and complex comorbidity profiles. It captures evolving clinical practice and highlights both the relevance and limitations of morphology-based embolic risk assessment in routine care. Our results reflect temporal changes in imaging utilization, microbiological profiles, and management strategies, which are often underrepresented in controlled or registry-based studies.

### 5.1. Embolic Burden in Contemporary IE Cohorts

Systemic embolization remains a frequent complication of IE and a major determinant of prognosis. Although cerebral and peripheral embolic events were globally balanced in this cohort, their distribution differed significantly according to vegetation phenotype. Mobile vegetations were predominantly associated with peripheral embolization, whereas patients without detectable vegetations presented almost exclusively with cerebral embolic events. This finding suggests that vegetation morphology may influence both embolic risk and anatomical distribution. Mobile vegetations, particularly those exceeding 10 mm, are exposed to repetitive shear stress during the cardiac cycle, increasing the likelihood of fragmentation and systemic embolization [2,8,10]. Conversely, cerebral embolic events that occur in the absence of detectable vegetations may reflect prior embolization with partial vegetation detachment or limitations in echocardiographic sensitivity [2,4,5]. The significant difference observed (*p* < 0.001) reflects variation in embolic territory rather than overall embolic frequency. Only 18.8% of cerebral embolic events were detected incidentally on imaging, likely reflecting limited access to systematic CT and especially MRI, before 2015. Consequently, the true burden of silent cerebral embolization may be underestimated in earlier cohorts [30,31,32]. These observations align with previous studies, demonstrating that embolic risk is highest early in the course of infection and declines after initiation of antibiotic therapy. This pattern supports early echocardiographic risk stratification [33,34].

### 5.2. Echocardiographic Predictors of Embolic Risk

Echocardiography remains central to embolic risk assessment in IE. Vegetations were more frequently identified in patients with embolic events, and both vegetation size and mobility were correlated with embolization. A vegetation length > 10 mm has consistently been associated with increased embolic risk and mortality [34,35], forming the basis for guideline recommendations regarding surgical timing [3,34,35]. However, size alone may inadequately capture emboligenic potential and is subject to interobserver variability [36]. Although embolic events were numerically more frequent in mitral IE, the difference compared to aortic involvement was not statistically significant, suggesting that embolic risk is more strongly influenced by vegetation morphology than by valve location. These findings support an increasing emphasis on dynamic morphological parameters that more accurately reflect emboligenic phenotype.

### 5.3. Vegetation Morphology as a Marker of Emboligenic Phenotype

Vegetation mobility has emerged as the most informative echocardiographic marker of embolic risk. Previous studies have demonstrated that highly mobile vegetations confer a substantially increased embolic risk, likely due to weaker attachment and greater exposure to hemodynamic shear forces [37,38]. In our cohort, mobile vegetations were strongly associated with embolization on both mitral and aortic valves, with particularly high odds ratios observed. Notably, all embolic events in aortic and multivalvular IE occurred in the presence of mobile vegetations.

Right-sided infective endocarditis was rare (three tricuspid cases). Given its distinct embolic pathways, its inclusion may introduce heterogeneity; however, the small number makes a meaningful impact unlikely. Separate or restricted analysis was not feasible and represents a limitation.

Vegetation mobility was assessed qualitatively using a consensus-based approach without formal reproducibility analysis. Although this represents a limitation, it reflects routine clinical practice, where mobility is visually assessed rather than quantitatively measured. While binary classification may oversimplify a dynamic phenomenon, it remains consistent with prior observational studies and guideline-based approaches. Accordingly, mobility should be interpreted as a pragmatic marker of emboligenic phenotype rather than a standardized quantitative predictor.

Embolic events occurred in 96 patients, while vegetations were detected in 75 cases. An important limitation is the discrepancy between embolic events (*n* = 96) and vegetation detection (*n* = 75). Some patients with embolic events had no detectable vegetations, possibly due to prior embolization, the limited sensitivity of TTE, or the presence of sub-resolution lesions [2,8,27]. This also has conceptual implications: morphology-based risk assessment applies only to patients with detectable vegetations and cannot fully capture embolic risk across the entire spectrum of infective endocarditis. Accordingly, vegetation characteristics should be interpreted as markers of embolic propensity in a subset of patients rather than as a universal predictive framework.

The interpretation of vegetation morphology in relation to embolic events should also consider the temporal heterogeneity of imaging practices throughout the study period. Although imaging strategies and TEE utilization evolved substantially during the study period, the association between vegetation morphology and embolic burden remained directionally consistent across study periods and according to embolic timing. These findings support the interpretation of vegetation morphology as a marker of an emboligenic phenotype rather than a strictly time-dependent predictor of future embolization [39,40].

Combined phenotype analyses showed that both vegetation size > 10 mm and mobility were consistently associated with embolic burden, supporting the relevance of vegetation morphology in embolic risk stratification [8,11,30,36]. Although mobility demonstrated numerically higher discriminatory performance, no statistically significant difference was observed between enhanced models. In addition, several subgroup estimates were affected by sparse-data bias and low event counts, limiting the precision of some effect estimates. Therefore, these findings should be interpreted cautiously and considered exploratory. Overall, the results support the value of a multiparametric echocardiographic assessment rather than reliance on a single vegetation characteristic.

### 5.4. Clinical Predictors of Embolic Events

In univariate analysis, CKD stage ≥ 3 was associated with embolic events. Renal dysfunction has been linked to systemic inflammation, endothelial dysfunction, and prothrombotic states that may increase susceptibility to embolization [40]. Female sex also emerged as an independent predictor, consistent with previous observations suggesting potential sex-related differences in inflammatory response or vegetation characteristics [41]. In multivariable analysis, increasing age was associated with a lower likelihood of embolization. Female sex remained positively associated with embolic risk, while DM showed an inverse association. The inverse association with diabetes is likely influenced by residual confounding, differences in comorbidity profiles, or limited statistical power, rather than representing a true protective effect. Similarly, the association observed for female sex should not be overinterpreted. Given the observational design, this finding may reflect differences in clinical presentation or underlying population structure rather than a specific biological mechanism. Both observations should therefore be considered hypothesis-generating and require confirmation in larger, prospective studies.

CKD ≥ stage 3 and reduced LVEF < 50% were not independently associated after adjustment. The loss of significance after adjustment suggests that CKD reflects overall disease severity rather than an independent embolic mechanism. The lack of independent association for LVEF indicates that global cardiac dysfunction plays a secondary role compared with vegetation-specific characteristics.

### 5.5. Role of Multimodality Imaging

Multimodality imaging has enhanced both the diagnostic and prognostic evaluation of IE. Echocardiography remains the first-line imaging modality, while CT, MRI, and PET/CT can improve the detection of extracardiac complications [42]. TEE offers higher sensitivity for detecting vegetations and perivalvular complications compared to TTE [43,44]. Emerging technologies, such as three-dimensional TEE, may further enhance morphological characterization and embolic risk stratification [45].

From a practical clinical perspective, not all valvular or intracardiac masses represent IE. The differential diagnosis includes thrombus, papillary fibroelastoma, nonbacterial thrombotic endocarditis, primary or metastatic cardiac tumors, and pseudomasses. Echocardiography provides the first-line imaging modality and provides the initial diagnostic orientation, allowing assessment of mass location, mobility, attachment, and associated valvular abnormalities. However, when findings are inconclusive or not fully concordant with clinical and microbiological data, a multimodal imaging approach is essential. This may include TEE for improved resolution, as well as complementary modalities such as cardiac CT, cardiac MRI, or PET/CT, particularly in complex cases or suspected prosthesis or CIED. Integrating imaging findings with clinical presentation and microbiological data is critical to avoid misclassification, especially in patients presenting with embolic events, where diagnostic accuracy directly impacts management decisions [46].

Beyond vegetation assessment, echocardiography plays a central role in evaluating suspected CIED-related infection. In contemporary practice, systematic imaging-particularly with TEE, when clinical suspicion persists-is essential for identifying lead-related masses, valvular involvement, and perivalvular extension. This is particularly relevant given the substantial proportion of CIED-related IE in the present cohort. Prevention of CIED infection remains a major unmet need and requires a structured approach, including risk assessment before device implantation and continued vigilance during follow-up. Emerging evidence supports both standardized prevention pathways and adjunctive strategies to reduce device-related infections, including targeted local anti-infective measures in selected patients [47,48]. These considerations highlight that TEE in this context extends beyond morphology assessment, playing a central role in the integrated management of device-related infection.

### 5.6. Arrhythmic Risk and Temporary Device Strategies in Infective Endocarditis

A further practical implication concerns patients with IE and transient arrhythmic risk, particularly those in whom permanent defibrillator implantation or reimplantation should be deferred due to active infection. In this context, temporary protection with a wearable cardioverter-defibrillator may represent a useful bridging strategy, allowing for the completion of antibacterial treatment, reassessment of left ventricular function, and more appropriate timing of definitive device decisions. Recent evidence supports a multiparametric approach to identify patients who may benefit from wearable protection while avoiding unnecessary permanent implantation, integrating clinical status, arrhythmic risk profile, and dynamic changes in cardiac function [49,50]. This approach may be particularly relevant in patients with cardiovascular implantable electronic devices (CIEDs), where reimplantation decisions are often complex and time-dependent.

### 5.7. Regional Perspective

Evidence from Eastern European populations remains limited and often reflects variability in access to cardiac surgery and advanced imaging. Observational studies from Romania have documented evolving epidemiology, including IVDU-associated IE and associated microbiological patterns. The relatively high rate of culture-negative IE likely reflects real-world referral patterns in a regional center, where many patients received empirical antibiotics before blood culture collection. This was particularly relevant during the earlier study period, when microbiological protocols and advanced diagnostic approaches were less standardized. Improved microbiological work-up and more systematic diagnostic strategies in the later period were associated with a lower proportion of culture-negative cases.

However, structured analyses of echocardiographic correlates of embolic burden remain limited [18,19,20]. Our findings provide additional real-world data from a regional referral center and support the clinical relevance of vegetation morphology within contemporary multiparametric risk assessment. In line with ESC 2023 recommendations, systematic echocardiographic evaluation may contribute to embolic risk stratification and clinical decision-making across heterogeneous healthcare settings [3].

### 5.8. Study Strengths and Limitations

Strengths of this study include a 15-year real-world cohort and detailed echocardiographic characterization, enabling the evaluation of combined phenotypes. Sequential multivariable modeling and ROC analysis quantified the incremental prognostic value of echocardiographic parameters. Limitations include the retrospective, single-center design and the potential underestimation of silent embolic events due to a lack of systematic imaging in earlier years. The relatively modest sample size in relation to the number of analyzed variables increases the risk of model overfitting and limits the stability of multivariable estimates. Incomplete information regarding prior outpatient antibiotic exposure limited the precise interpretation of culture-negative cases.

The extended study period introduced temporal heterogeneity related to evolving imaging practices, microbiological diagnostics, and management strategies. TEE utilization increased progressively over time and was not systematically available in the earlier period, which may have introduced verification bias and influenced vegetation detection and characterization, particularly in clinically higher-risk patients. However, sensitivity analyses across time periods demonstrated consistent associations, suggesting that the main findings are robust despite these secular changes.

The statistical analysis has several limitations inherent to the retrospective design, including possible residual confounding and evolving imaging strategies over time. Sparse-data bias in some subgroup analyses reduced the precision of several odds ratio estimates, particularly in low-frequency phenotype comparisons. In addition, no internal validation or calibration analysis was performed; therefore, the observed improvement in discrimination after inclusion of echocardiographic parameters should be interpreted cautiously and considered exploratory rather than a validated predictive model. Future studies employing prospective designs, standardized imaging protocols, and validated multivariable models are needed to confirm the study findings.

### 5.9. Clinical Implications

In routine clinical practice, vegetation morphology may provide complementary information in the assessment of patients with IE, particularly when integrated with clinical severity, microbiological profile, and cardiac complications. Larger and mobile vegetations were more frequently associated with embolic burden in this cohort, supporting their inclusion in a broader multiparametric evaluation rather than as isolated decision-making criteria.

Given the retrospective design, variability in imaging strategies, and absence of standardized time-dependent assessment, these findings should not be interpreted as establishing validated predictive thresholds. Instead, they support the practical value of careful echocardiographic characterization as part of individualized clinical assessment and multidisciplinary decision-making in patients with IE.

### 5.10. Future Directions

Prospective multicenter studies are needed to validate mobility-based risk models. Standardization of vegetation morphology, especially mobility assessment, may improve reproducibility. Integrating echocardiographic morphology with clinical, microbiological, and multimodality imaging parameters may further refine risk stratification. Emerging artificial intelligence approaches may enable automated quantification of vegetation characteristics, thereby improving objective embolic risk assessment.

## 6. Conclusions

Embolic events were frequent in this real-world cohort and reflected the overall in-hospital embolic burden. Vegetation mobility and size > 10 mm were independently associated with embolic events, supporting their role as markers of an emboligenic phenotype in patients with detectable vegetations. Given the observational design and methodological limitations, these findings should be interpreted as associative rather than predictive and require prospective validation.

**Key Messages**

- Systemic embolization remains a frequent complication of IE in real-world clinical practice.
- Embolic burden was consistently associated with vegetation morphology, particularly vegetation mobility and size > 10 mm.
- Vegetation mobility and size may provide complementary information within a multiparametric embolic risk assessment.
- Careful echocardiographic characterization of vegetations may support individualized clinical evaluation and multidisciplinary decision-making in selected patients with IE.

## Figures and Tables

**Table 1:** Baseline Characteristics and In-Hospital Outcomes According to Embolic Event Status.

Variable	Overall (*n* = 164)	No Embolic Events (*n* = 68)	Embolic Events (*n* = 96)	*p*-Value Difference (95% CI)
Demographics				
Age, years (mean ± standard deviation)	64 ± 12.7	66 ± 12.6	62 ± 12.2	0.032 (−9.5 to −1.0)
Male sex, *n* (%)	113 (68.9%)	52 (76.5%)	61 (63.5%)	0.112
Comorbidities				
Hypertension, *n* (%)	101 (61.6%)	46 (67.6%)	55 (57.3%)	0.238
Diabetes mellitus, *n* (%)	26 (15.9%)	16 (23.5%)	10 (10.4%)	0.041 (−0.01 to 0.25)
CKD (≥stage 3), *n* (%)	24 (14.6%)	5 (7.4%)	19 (19.8%)	0.046 (0.02 to 0.23)
IVDU, *n* (%)	4 (2.4%)	0 (0.0%)	4 (4.2%)	0.142 *
Chronic HF, *n* (%)	117 (71.3%)	48 (70.6%)	69 (71.9%)	0.997
Liver cirrhosis, *n* (%)	5 (3.0%)	3 (4.4%)	2 (2.1%)	0.650
Malignancy, *n* (%)	27 (16.5%)	15 (22.1%)	12 (12.5%)	0.158
Infection location				
Left-sided IE, *n* (%)	130 (79.3%)	58 (85.3%)	72 (75.0%)	0.160
Right-sided IE, *n* (%)	5 (3.0%)	0 (0.0%)	5 (5.2%)	0.077 * (0.01 to 0.10)
Prosthetic IE, *n* (%)	68 (41.5%)	34 (50.0%)	34 (35.4%)	0.088 (−0.30 to 0.01)
CIED-related IE, *n* (%)	25 (15.2%)	11 (16.2%)	14 (14.6%)	0.953
TAVI-associated IE, *n* (%)	8 (4.9%)	1 (1.5%)	7 (7.3%)	0.141
Microbiology				
*S. aureus*, *n* (%)	22 (13.4%)	9 (13.2%)	13 (13.5%)	1.000
*Enterococcus* spp., *n* (%)	24 (14.6%)	13 (19.1%)	11 (11.5%)	0.253
Viridans streptococci, *n* (%)	6 (3.7%)	1 (1.5%)	5 (5.2%)	0.402
Culture-negative IE, *n* (%)	77 (47.0%)	32 (47.1%)	45 (46.9%)	1.000
Outcomes				
Acute HF, *n* (%)	18 (11.0%)	7 (10.3%)	11 (11.5%)	1.000
Septic shock, *n* (%)	9 (5.5%)	6 (8.8%)	3 (3.1%)	0.165
Cardiogenic shock, *n* (%)	26 (15.9%)	11 (16.2%)	15 (15.6%)	1.000
Renal replacement therapy	6 (3.7%)	2 (2.9%)	4 (4.2%)	1.000
Surgery performed, *n* (%)	45 (27.4%)	14 (20.6%)	31 (32.3%)	0.140
Appropriate antibiotic therapy, *n* (%)	106(64.7%)	43(62.6%)	63(65.6)	0.588.
In-hospital mortality	53 (32.3%)	17 (25.0%)	36 (37.5%)	0.129
Length of stay, days (mean ± standard deviation)	8 ± 9.6	10 ± 9.8	8 ± 8.9	0.697

Legend: CKD = chronic kidney disease was defined as estimated glomerular filtration rate < 60 mL/min/1.73 m^2^ (stage ≥ 3) or need for chronic dialysis; Chronic HF = existing heart failure documented before the index hospitalization; IE = infective endocarditis; IVDU = intravenous drug use; CIED = cardiac implantable electronic device; TAVI = transcatheter aortic valve implantation; Acute HF on admission = acute decompensated heart failure present at hospital admission; *n* = number; %—percentages; Difference (95% CI/confidende interval) represents the absolute difference in proportions between embolic and non-embolic groups; *p*-value < 0.05 was considered statistically significant; * = for variables with small cell counts; Fisher’s exact test was used; Difference CI for proportions should be interpreted cautiously when zero cells are present.

**Table 2:** Distribution of embolic territory across vegetation phenotypes.

Vegetation Phenotype	Cerebral Embolism–Symptomatic	Cerebral Embolism–Imaging Detected	Peripheral Embolism	Total Embolic Events	Proportion of Total Embolic Events (%)
Mobile vegetation > 10 mm (*n* = 52)	11 (21.2%)	6 (11.5%)	35 (67.3%)	52	54.1%
Mobile vegetation ≤ 10 mm (*n* = 22)	4 (18.2%)	5 (22.7%)	13 (59.1%)	22	22.9%
Non-mobile vegetation (*n* = 1)	0 (0%)	0 (0%)	1 (100%)	1	1.04 *
No vegetation detected (*n* = 21)	14 (66.7%)	7 (33.3%)	0 (0%)	21	21.9%
Total embolic events (*n* = 96)	29 (30.2%)	18 (18.8%)	49 (51.0%)	96	100
Statistical comparisons Distribution of embolic territory across vegetation phenotypes (chi-square test); cerebral vs. peripheral embolism across vegetation phenotypes; *p* < 0.001 ** Vegetation size > 10 mm vs. ≤10 mm; *p* = 0.49					

Legend: Among the 96 patients with embolic events, echocardiographic vegetations were identified in 75 patients, while 21 patients had no vegetation detected at the time of echocardiographic examination; cerebral embolic events included both clinically symptomatic ischemic stroke and silent cerebral infarctions detected by neuroimaging during preoperative assessment. Peripheral embolic events comprised embolization to splenic, renal, hepatic, mesenteric, or limb arteries; percentages represent the distribution of embolic events within each phenotype; statistical comparisons refer to distribution differences, not absolute embolic risk. * Estimates for this subgroup are not reliable due to extremely small sample size; ** the reported *p*-value refers to the comparison of embolic territory distribution across vegetation phenotypes, rather than to the global proportion of cerebral versus peripheral embolic events; although the global proportion of cerebral and peripheral embolic events was similar (49% vs. 51%), their distribution differed significantly across vegetation phenotypes.

**Table 3:** Echocardiographic Characteristics According to Embolic Event Status.

Echocardiography Variables	Overall (*n* = 164)	No Embolic Events (*n* = 68)	Embolic Events (*n* = 96)	*p*-Value Difference (95% CI)
Vegetation present, *n* (%)	112 (68.3%)	37 (54.4%)	75 (78.1%)	0.002 (0.09 to 0.38)
Vegetation, mm (mean ± standard deviation)	12.13 ± 2.68 (11.72–12.54)	14 ± 0.5 (13.9–14.1)	9.5 ± 0.5 (9.38–9.62)	0.0001 (4.04 to 4.96)
Vegetation > 10 mm, *n* (%)	74 (45.1%)	14 (20.6%)	60 (62.5%)	<0.001 (0.28 to 0.56)
Vegetation mobility	93 (56.7%)	19 (27.9%)	74 (77.1%)	<0.001 (0.3 to 0.69)
Vegetation mobility and >10 mm	67(40.8%)	14(20.5%)	53(55.2%)	<0.001 (0.18 to 0.5)
Mitral vegetations (native and prosthesis), *n* (%)	47(42%)	15(40.5%)	32(42.6%)	0.16
Aortic vegetations (native and prosthesis), *n* (%)	44(39.2%)	17(45.9%)	27(36%)	0.72
Tricuspid vegetations (native), *n* (%)	3 (2.7%)	0	3 (4%)	0.27 *
Multivalvular vegetations (native and prosthesis), *n* (%)	18(16.07%)	3(8.1%)	15(20%)	0.024
Perivalvular abscess, *n* (%)	38 (23.2%)	17 (25.0%)	21 (21.9%)	0.780
Severe valvular regurgitation, *n* (%)	55 (33.5%)	20 (29.4%)	35 (36.5%)	0.439
LVEF < 50%, *n* (%)	72 (43.9%)	23 (33.8%)	49 (51.0%)	0.042 (0.02 to 0.32)

Legend: Multivalvular = aortic and mitral, LVEF = left ventricular ejection fraction, *n* = number, mm = millimeters, % = percentages, Difference (95% CI/confidence intervals) represents the absolute difference in proportions between embolic and non-embolic groups; estimates in small subgroups (e.g., tricuspid vegetations) should be interpreted with caution due to limited sample size. * No statistical inference possible due to small sample size; *p*-value < 0.05 was considered statistically significant.

**Table 4:** Combined Echocardiographic Phenotype and Embolic Risk in patients with identified vegetations.

Vegetation Phenotype	No Embolic (*n*)	Embolic (*n*)	Total (*n*)	Embolic Proportion (%)	OR (95% CI)	*p* Value
Non-mobile vegetation ≤ 10 mm, (*n*)	18	2	20	10%	1.00	—
Mobile vegetation ≤ 10 mm, (*n*)	4	22	26	84.6%	49.5 (5.5–440) ***	<0.001
Mobile vegetation > 10 mm, (*n*)	14	52	66	78.8%	33.4 (4.5–250) ***	<0.001
Mobile > 10 mm vs. Mobile ≤ 10 mm, (*n*)	14 vs. 4	52 vs. 22	66 vs. 26	78.8% vs. 84.6%	0.67 (0.47–4.96)	0.5
Valve-specific analysis						
Mitral vegetations non mobile vs. mobile, (*n*)	7 vs. 8	1 vs. 31	47	68.1%	27.1 (2.9–253.4) ***	0.0007
Aortic vegetations non mobile vs. mobile, (*n*) *	9 vs. 8	0 vs. 27	44	61.4%	61.5 (3.2–1180) ***	<0.001
Multivalvular non mobile vs. mobile, (*n*) **	2 vs. 3	0 vs. 13	18	72.2%	17.3 (0.75–400) ***	0.06 **
Overall vegetations non mobile vs. mobile, (*n*)	18 vs. 19	1 vs. 74	112	66.9%	37 (7.96–172.1) ***	<0.001

Legend: *n* = number, mm = millimeters, % = percentages, OR = odds ratio, 95% CI = 95% confidence intervals, *p*-value < 0.05 was considered statistically significant. * In this cohort, all embolic events in aortic valve infective endocarditis occurred in patients with mobile vegetations, ** in multivalvular vegetations locations (aortic and mitral), all embolic events occurred in patients with mobile vegetations (13 vs. 0), showing a strong trend toward association (Fisher *p* = 0.065), *** wide confidence intervals reflect sparse data and zero-cell corrections, OR for subgroups with zero cells were estimated using Haldane–Anscombe correction-estimates should be interpreted cautiously due to small subgroup sizes.

**Table 5:** Univariable Analysis of Predictors of Embolic Events.

Variable	OR	95% CI	*p*
Clinical			
Age (per 10 years)	0.81	0.65–1.01	0.065
Female sex	1.86	0.93–3.75	0.080
Diabetes mellitus	0.38	0.16–0.89	0.027
CKD ≥ stage 3	3.32	1.18–9.34	0.023
Echocardiographic			
Mobile vegetations	8.67	4.26–17.68	<0.001
Vegetation > 10 mm	6.43	3.13–13.9	<0.001
Reduced LVEF < 50%	2.13	1.12–4.04	0.021

Legend: CKD = chronic kidney disease was defined as estimated glomerular filtration rate < 60 mL/min/1.73 m^2^ (stage ≥ 3) or need for chronic dialysis, LVEF = left ventricular ejection fraction, *n* = number, mm = millimeters, %—percentages, 95% CI = 95% confidence intervals, *p*-value < 0.05 was considered statistically significant.

**Table 6:** Multivariable Logistic Regression Models and Discriminative Performance for Embolic Events.

Variable	Model A OR (95% CI)	*p*	Model B1 OR (95% CI)	*p*	Model B2 OR (95% CI)	*p*
Age (per 10 years)	0.98 (0.96–1.00)	0.065	0.74 (0.60–0.90)	0.028	0.74 (0.6–0.9)	0.031
Female sex	1.82 (0.96–3.46)	0.067	2.11 (1.08–4.13)	0.029	2.05 (1.05–4.02)	0.034
Diabetes mellitus	0.41 (0.17–0.99)	0.048	0.38 (0.15–0.94)	0.036	0.40 (0.16–0.96)	0.041
CKD ≥ 3	2.84 (1.01–7.96)	0.047	2.65 (0.94–7.42)	0.064	2.71 (0.97–7.59)	0.059
LVEF < 50%	1.89 (0.98–3.66)	0.058	1.76 (0.91–3.40)	0.089	1.81 (0.94–3.5)	0.076
Vegetation mobility	—	—	3.62 (1.75–7.48)	<0.001	—	—
Vegetation > 10 mm	—	—	—	—	3.48 (1.69–7.16)	<0.001
Calculated AUC	0.71		0.81		0.79	

Legend: Model A: clinical variables, Model B1: Model A + vegetation mobility, Model B2: Model A + vegetation size > 10 mm, CKD = chronic kidney disease was defined as estimated glomerular filtration rate < 60 mL/min/1.73 m^2^ (stage ≥ 3) or need for chronic dialysis, LVEF = left ventricular ejection fraction, *n* = number, mm = millimeters, %—percentages, OR = odds ratio, 95% CI = 95% confidence intervals, *p*-value < 0.05 was considered statistically significant.

## Data Availability

The data underlying this study were collected as part of routine clinical care and analyzed retrospectively. Due to institutional and ethical restrictions related to patient confidentiality, the dataset is not publicly available. De-identified data may be made available from the corresponding author upon reasonable request and subject to institutional approval.
